# GEJ Adenocarcinoma Following Antireflux Surgery: A Missed Diagnosis

**DOI:** 10.14309/crj.0000000000001883

**Published:** 2025-10-30

**Authors:** Christine Son, Muneeb Ansari, Sally Ceesay, Joshua Kalapala, Joyce Loh, Hao Zhang

**Affiliations:** 1 Internal Medicine, Loyola University Medical Center, Maywood, IL; 2 Gastroenterology and Hepatology, Loyola University Medical Center, Maywood, IL; 3 Loyola University Chicago Stritch School of Medicine, Maywood, IL; 4 Northwestern University Feinberg School of Medicine, Chicago, IL

**Keywords:** gastroesophageal junction adenocarcinoma, antireflux surgery, Barrett's esophagus

## Abstract

Adenocarcinoma of the gastroesophageal junction is a rare condition with no specific screening guidelines. While fundoplication is the surgical treatment of choice for gastroesophageal reflux disease, many patients continued to have recurrent symptoms postprocedure with several structural complications after. Antireflux surgery does not prevent esophageal adenocarcinoma. We present a case of a 76-year-old woman with a history of severe gastroesophageal reflux disease and hiatal hernia treated with repeat fundoplication who presented with progressively and rapidly worsening dysphagia to solids and liquids. The patient was found to have a poorly differentiated adenocarcinoma at the gastroesophageal junction, when the esophagogastroduodenoscopy 2 months had missed the diagnosis.

## INTRODUCTION

Adenocarcinoma of the gastroesophageal junction (GEJ) is a rare condition with no specific screening guidelines.^1^ The majority of adenocarcinoma of the esophagus and GEJ arise from Barrett's esophagus (BE) with gastroesophageal reflux disease (GERD) and obesity as major risk factors. However, less than 10% of patients with GEJ adenocarcinoma had known BE.^2^ A study in 2011 showed that treating GERD with antireflux surgery does not prevent esophageal adenocarcinoma, while BE and endoscopic esophagitis remain the main risk factors.^3^

While fundoplication is considered the surgical treatment of choice for GERD, up to 30% of patients have recurrent symptoms after the procedure, such as dysphagia and dyspepsia.^4^ Structural complications at the boundary between the esophagus and cardia arise due to mechanical stress, which makes the fundoplication wraps susceptible to disruption, herniation, or slippage.

## CASE REPORT

**Figure 1.**
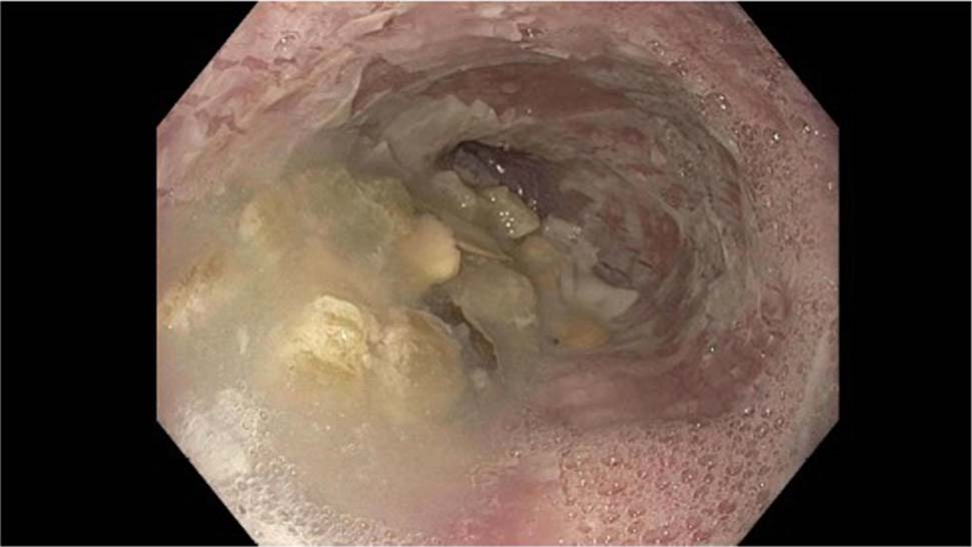
Food was found in the middle third of the esophagus and lower third of the esophagus, suggesting stasis and abnormal motility.

**Figure 2.**
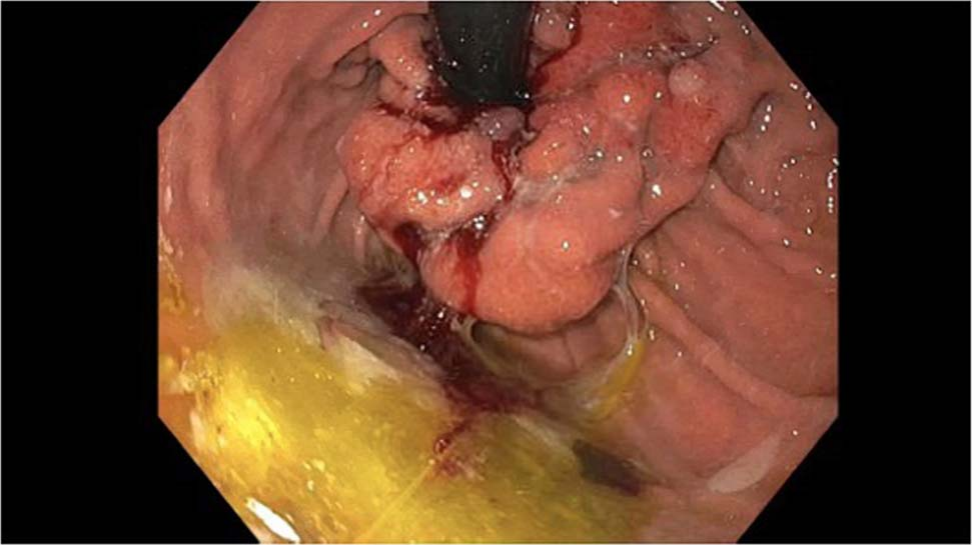
Localized severe mucosal changes characterized by erythema, friability (with spontaneous bleeding), inflammation, and ulceration concerning for malignancy were found in the gastric cardia. Multiple biopsies were taken for histology.

A 76-year-old woman with a history of severe GERD and hiatal hernia treated with a Nissen fundoplication in 2000 and Toupet fundoplication in 2014 presented to an outside hospital with progressively rapidly worsening dysphagia to solids and liquids. The patient underwent esophagogastroduodenoscopy (EGD) 2 months before presentation which showed achalasia vs stricture and underwent dilation with improvement of symptoms. A week later, the patient was transferred to our institution for further care. The patient underwent repeat EGD which showed moderate food debris in the middle and lower esophagus and ulcerated, nodular-appearing mucosa at the GEJ, and gastric cardia (Figures 1 and 2). Biopsies are consistent with poorly differentiated adenocarcinoma. The patient underwent endoscopic ultrasound (EUS)/EGD that showed a partially obstructing esophageal tumor in the distal esophagus and multiple nodules found in the stomach. The patient was diagnosed with malignant gastric adenocarcinoma at the GEJ staged T2 N1 M0. Positron emission tomography computed tomography showed no evidence of distant metastasis but increased metabolic activity in the proximal stomach consistent with the diagnosis. The patient is currently undergoing discussion to proceed with fluorouracil, leucovorin, oxaliplatin, and docetaxel (FLOT)-based neoadjuvant chemotherapy followed by restaging scans for possible curative resection pending clinical course.

## DISCUSSION

The patient was diagnosed with esophageal motility disorder via manometry several years following her first Nissen fundoplication in 2000 prompting repeat fundoplication in 2014. Before her repeat fundoplication in 2014, EGD evaluation in 2010 showed intact fundoplication and showed normal appearing esophagus and intact fundoplication wrap. No esophagitis, intestinal metaplasia, or BE noted. The patient had persistent symptoms, prompting repeat EGD evaluation in 2012, which showed the same findings. However, she was empirically dilated due to her symptoms of dysphagia and abnormal esophagram that revealed a very narrowed GEJ causing retention of barium and likely food within distal dilated esophagus. There was no evidence of esophageal peristaltic wave and mild esophageal spasm was identified. Her persistent dysphagia led to repeat EGD in 2013, which showed fluid in the lower third of the esophagus likely related to hiatal hernia and slipped fundoplication. Esophageal mucosa was otherwise unremarkable without active esophagitis, rings, strictures, or evidence of Barrett's. Manometry at the time showed significant esophageal dysmotility. Peristalsis was present, but only with 60% of swallows. There were 10% that were simultaneous and 30% that were failed. There were no double or triple-peak contractions. Lower esophageal sphincter resting pressure was 17.2 mm Hg, with normal range being 13 up to 43 mm Hg. Owing to these findings, the patient was recommended laparoscopic conversion of her Nissen to a partial, Toupet fundoplication in 2014.

The progression of the patient's symptoms over the years and changes evident of stricture and/or achalasia 2 months before presentation indicates failure of repeat fundoplication and some presenting factors interfering with the mucosal defense system that is not prevented by fundoplication.

One theory behind the increased susceptibility to a protumor environment following an antireflux procedure is retention esophagitis. Retention esophagitis is the process in which chronic stasis of food or liquid in the esophagus leads to inflammation, ulceration, and changes to the esophageal lining. It is a notable endoscopic finding with the presence of a thickened or whitish esophageal mucosa and altered squamous hyperplasia seen histologically.^5^ Retention esophagus is closely associated with achalasia, which is thought to be a premalignant lesion associated with increased risk of esophageal cancer.^5^

A population-based study in 2011 looked at the incidence of esophageal adenocarcinoma after antireflux surgery.^3^ Their results showed a 9.2 hold incidence of esophageal adenocarcinoma in patients treated with antireflux surgery compared with that of the general population. While patients undergoing antireflux procedures may have higher malignancy risk at baseline, this study showed the need for long follow-up times following antireflux surgery as most esophageal adenocarcinomas developed very late after the surgery, similar to our patient.

Previous studies also showed findings supportive of carcinogenic environments in post-op patients on the molecular level. The study by Rantanen et al demonstrated higher proliferation activity makers with Ki-67 and Bcl-2 expression in the GEJ and the distal and proximal esophagus at 48 months following antireflux surgery, supporting the hypothesis that there may be increased proliferative activity after fundoplication in the distal esophagus despite a normal fundic wrap and healing of GERD.^6^ In addition, heat shock proteins (hsps), which have a role in promoting tumorigenesis by inhibiting apoptosis in the pathogenesis of GERD, as well as the malignant transformation of BE, may be uninfluenced by fundoplication, as there were no differences in expression of hsp27 and hsp70 in the EGJ preoperatively and postoperatively.^7^ The presence of some factor interfering with the mucosal defense system of the distal esophagus in GERD may be unaffected by fundoplication and not associated with the acid-reflux-normalizing effect.

This case also warrants attention to the trends of post endoscopy esophageal neoplasia (PEEN) and post endoscopy esophageal adenocarcinoma (PEEC). PEEN and PEEC refer to high grade dysplasia or esophageal adenocarcinoma detected before the next recommended surveillance endoscopy in a patient with nondysplastic BE.^8^ Possible explanations for PEEN/PEEC cases are possible missed visible lesion from inadequate previous examination, incomplete resection of previously identified lesion, and patient failing to follow-up on a recommended surveillance endoscopy.^8^ One case report demonstrated that patient with GERD and BE was diagnosed with adenocarcinoma of lower esophagus 8 years following the fundoplication.^9^ One population study in 2011 in Finland looked at frequency and predisposing factors for post-fundoplication esophageal adenocarcinoma.^3^ Common preoperative findings were esophagitis (77%), hiatal hernia (75%), BE (45%), and ulcer in the GE junction (17%), and strictures (6%) in the study.^9^ The antireflux surgery had preceded cancer diagnosis at a mean interval of 10.1 years, similar to our patient. This shows that patients with not only preoperative BE but other findings like esophagitis and hiatal hernia need close and long-term endoscopic and histologic surveillance because dysplasia and/or adenocarcinoma can appear at later follow-up.

This case is unique as the EGD performed 2 months before presentation missed the cancer diagnosis as the mass was hidden within the folds of previous fundoplication. The absent peristalsis in a tight lower esophageal sphincter was suggestive of either a primary esophageal dysmotility or a sequelae of patient's previous fundoplication. In patients who later develop persistent dysphagia symptoms, careful endoscopic evaluation in the gastric cardia around wrap is important. For suspected mucosal abnormalities, biopsies are always recommended. This case report also seeks the need for further investigation on whether patients who have undergone fundoplication should be screened more regularly with a longer follow-up for adenocarcinoma. Malignancy should always remain on the differential even with previous negative endoscopy.

## DISCLOSURES

Author contributions: C. Son drafted the manuscript and approved the final draft submitted. M. Ansari drafted the manuscript and approved the final draft submitted. S. Ceesay performed literature search. J. Kalapala performed literature search. J. Loh has overseen the case and provided critical review and edits to the manuscript. H. Zhang has overseen the case and provided critical review and edits to the manuscript and is the article guarantor.

Acknowledgments: We thank the department of Gastroenterology's nursing and staff at Loyola University Medical Center for their cooperation with the case.

Financial disclosure: None to report.

Informed consent was obtained for this case report.

## ABBREVIATIONS:

BE: Barrett's esophagus
EGD: esophagogastroduodenoscopy
EUS: endoscopic ultrasound
FLOT: fluorouracil leucovorin oxaliplatin and docetaxel
GEJ: gastroesophageal junction
GERD: gastroesophageal reflux disease
Hsp: heat shock proteins
PEEN: post-endoscopy esophageal neoplasia
PEEC: post-endoscopy esophageal adenocarcinoma
